# Adoptive transfer of tumor infiltrating lymphocytes for metastatic cervical cancer

**DOI:** 10.1186/2051-1426-1-S1-P15

**Published:** 2013-11-07

**Authors:** Christian S Hinrichs, Sanja Stevanovic, Lindsey Draper, Michelle Langhan, Mark Dudley, John Wunderlich, Steven A Rosenberg

**Affiliations:** 1 National Cancer Institute - Surgery Branch, Bethesda, MD, USA

## 

Adoptive T-cell therapies for cancer can induce tumor responses in patients with metastatic melanoma, synovial sarcoma, and B-cell malignancies. However, no cellular therapy has demonstrated activity in a common epithelial tumor. Adoptive transfer of tumor infiltrating lymphocytes (TIL) has high response rates and can induce complete and durable tumor regression in metastatic melanoma. We sought to determine the objective tumor response rate of TIL therapy for human papillomavirus (HPV)-associated malignancies. These common epithelial cancers occur at varied sites but universally express the immunogenic E6 and E7 viral oncoproteins making them rational targets for T cell-based therapeutic approaches. Herein we report the results of treatment of the first eight subjects (seven evaluable) from the cohort of patients with cervical cancer (which is necessarily associated with high-risk HPV infection) treated with TIL therapy. Tumors were HPV genotyped and TIL were generated from individual tumor fragment cultures then tested for reactivity against autologous dendritic cells loaded with pools of overlapping peptides spanning type-specific HPV E6 or E7 proteins. Cultures were selected for rapid expansion and administration to patients based on oncoprotein reactivity, T-cell growth rate, and CD8+ T cell frequency. Patients received non-myeloablative lymphoconditioning chemotherapy before cell infusion and high-dose bolus IL-2 after cell infusion. TIL from 6/8 patients displayed reactivity against E6 or E7. Objective tumor responses by RECIST criteria were observed in 3/7 evaluable patients; two have complete and ongoing regression of measurable disease at three and nine months after treatment. Adverse events were the expected combined toxicities of the preparative chemotherapy regimen and interleukin-2, and were consistent with those of TIL therapy for metastatic melanoma. No autoimmunity was observed. These early clinical trial results indicate that tumor responses can occur following administration of TIL for metastatic cervical cancer.

